# Exposure to manganese during juvenile development increases microglial activation in the hippocampus following systemic infection with A/California/04/2009 Influenza A H1N1 virus

**DOI:** 10.3389/ftox.2026.1789730

**Published:** 2026-04-02

**Authors:** Megan R. Hager, Adam J. Schuller, Omar A. Yanouri, Collin M. Bantle, Richard J. Smeyne, Ronald B. Tjalkens

**Affiliations:** 1 Department of Environmental and Radiological Health Sciences, Colorado State University, Fort Collins, CO, United States; 2 Department of Neuroscience, Vickie & Jack Farber Institute for Neuroscience, Thomas Jefferson University, Philadelphia, PA, United States

**Keywords:** hippocampus, immune priming, manganese, microglia, neurodegeneration, neuroinflammation, neurotoxicology, viral infection

## Abstract

Up to 80% of patients with Parkinson’s Disease (PD) develop dementia within 20 years of diagnosis. Although the etiology of PD and related neurodegenerative disorders is poorly understood, risk factors including environmental toxicants and viral infections are linked to disease onset and progression. Exposure to high doses of the essential element, manganese (Mn), causes neurotoxicity associated with parkinsonian symptoms and cognitive impairment in humans. Additionally, epidemiologic studies indicate that viral infections increase risk of developing PD. Previously, our lab demonstrated that mice exposed to Mn during juvenile development showed greater neuroinflammatory changes in microglia within the substantia nigra following systemic infection with H1N1 influenza virus (California/04/09 influenza A) than mice infected without prior exposure to Mn. In the present study, this murine dual-hit model was employed to investigate how juvenile Mn exposure alters H1N1-induced neuropathology and glial morphology in the hippocampus. Mice were exposed to Mn in drinking water from post-natal day 21–51 and then intranasally infected with 10^3^ TCID_50_ A/California/04/2009 H1N1. To assess histopathology following this exposure paradigm, we performed high-content microscopy and machine learning-based image analysis of H&E and IHC-stained sections spanning the hippocampus to quantify pyknotic neurons and reactive microglia. We report a significant increase in the number of pyknotic neurons in the dentate gyrus as well as morphologic changes in microglia that are consistent with inflammatory activation. Our findings highlight the capacity of combined juvenile manganese exposure and adult viral infection to induce substantial microgliosis in the hippocampus.

## 1 Introduction

Parkinson’s Disease (PD) is a progressive, age-related movement disorder that is rapidly increasing in prevalence worldwide (Dorsey et al., 2018). PD is characterized by the selective loss of dopaminergic neurons in the substantia nigra pars compacta (SNpc) and pathologic aggregation of alpha-synuclein, resulting in well-defined symptoms of motor dysfunction (Bloem et al., 2021). Interestingly, some reports indicate that up to 80% of PD patients go on to develop dementia within 20 years of diagnosis (Hely et al., 2008). A variety of terms exist to classify this phenomenon under an expanding umbrella of “synucleinopathies” including Lewy Body Dementia (LBD), which encompasses Parkinson’s Disease Dementia (PDD) and dementia with Lewy Bodies (DLB) (Lau et al., 2025). Increasing evidence links environmental toxicant exposure with the incidence of age-related neurodegeneration, including PD and LBD (Lau et al., 2025).

Manganese (Mn) is an essential trace mineral that can cause an extrapyramidal form of parkinsonism termed manganism following excessive accumulation in the basal ganglia. This movement disorder is characterized by motor symptoms that include bradykinesia and dystonia, as well as cognitive deficits and emotional lability (Calne et al., 1994). Chronic Mn exposure has also been shown to increase the longitudinal risk of developing PD (Lucchini and Tieu, 2023). Children who are exposed to excessive Mn in their drinking water exhibit deficits in cognitive performance and verbal function (Bjorklund et al., 2017), which may result from heightened synaptic pruning by overactive microglia (Tjalkens et al., 2017; Verina et al., 2011; Lao et al., 2017). Our group has shown that juvenile mice are more sensitive to the neurologic effects of Mn overexposure than adult mice (Moreno et al., 2009a), and that changes during this critical window of development can prime adult susceptibility to secondary insults (Moreno et al., 2009b; Bantle et al., 2021a). Additionally, a growing body of work has implicated systemic viral infection as a contributor to the onset of PD. Neurotropic viruses have been shown to directly induce dopaminergic neuron loss in the SNpc (Bantle et al., 2019; J et al., 2012; Bantle et al., 2021b). Non-mouse adapted H1N1 CA/09, a non-neurotropic virus, has also been reported to elicit a state of pro-inflammatory activation in microglia (Sadasivan et al., 2015). Another non-neurotropic Puerto Rican strain of H1N1 has additionally been shown to increase proportional Iba1+ area and alter dentate neuron morphology (Jurgens et al., 2012). These reports highlight the importance of studying a variety of environmental insults across the exposome in the context of immune modulation, glial-mediated neuroinflammatory signaling, and neurodegenerative disease onset and progression.

Innate immune memory has been postulated as a mechanism of inflammatory potentiation across body systems, with special importance in the brain parenchyma given the emergence of neuroinflammation as a key mechanism shared by many neurodegenerative disorders (Lefevre-Arbogast et al., 2024; Zhang et al., 2023). Supporting this, a variety of studies have emerged demonstrating that sequential exposure to systemic immune modulatory insults can alter neuroinflammatory and neurotoxic outcomes (Bantle et al., 2021a; Purisai et al., 2007; Sadasivan et al., 2017; Hammond et al., 2020). Microglia are resident immune cells in the brain and, like peripheral macrophages, arise from embryonic yolk sac origins (Li and Barres, 2018). These cells play a key role in an array of critical functions, such as myelination (McNamara et al., 2023; Chen et al., 2025), synaptic pruning (Paolicelli et al., 2011), and neuroinflammation (Li and Barres, 2018). Aberrant proliferation and activation of microglia can result in dysregulation of these necessary functions in the central nervous system. Still, little work has been done to investigate the effects of serial environmental toxicant exposure on hippocampal histopathology, a brain region critical to cognitive and executive function. This motivated our present application of a dual-hit murine model of heavy metal exposure and systemic viral infection to investigate the combined effects of pro-inflammatory stressors. Accordingly, we here exposed juvenile mice to Mn via drinking water and subsequent intranasal infection to probe microglial reactivity and neuronal density using cutting-edge techniques in digital pathology, including high content microscopy and deep learning-based image analysis. Increased characterization of the etiological risk factors associated with neurodegenerative disease onset and progression will further our ability to diagnose and treat these conditions with more nuanced understanding of the interplay between different environmental risk factors for disease.

## 2 Methods

### 2.1 Exposure and infection protocol

Colorado State University and St. Jude Children’s Hospital Institutional Animal Care and Use Committees (IACUCs) approved all animal procedures prior to conducting experiments which proceeded in compliance with all National Institute of Health guidelines. C57Bl/6 mice were acquired from Jackson Laboratory and housed in breeder pairs in a temperature-controlled room (maintained at 22 °C–24 °C) on a 12 h light/dark cycle with *ad libitum* access to standard mouse chow. A detailed description of the exposure protocol can be found in Dorsey and Bloem (2024). In short, starting at day PND21, C57BL/6 mice were given MnCl_2_ (50 mg/kg/day; Sigma) or normal drinking water for 30 days. All mice were monitored for normal weight gain and water consumption. At PND51, all mice were restored to regular drinking water for 1 month. Mice were then intranasally infected with 10^3^ TCID_50_ A/California/04/2009 (CA/09) H1N1 or mock-infected with 25 μL saline (*n* = 3-4 per group), as described (Sadasivan et al., 2015). Daily monitoring for 21 days post-infection ensured no overt neurobehavioral abnormalities or clinical signs of moribundity. Mice maintained consistent bodyweight and experienced no mortality. After 21 days, all mice were euthanized under deep Avertin anesthesia, transcardially perfused with 3% paraformaldehyde, and brains were collected and post-fixed in the same fixation buffer.

### 2.2 Tissue processing, staining, and imaging

Following fixation, all brains were processed for paraffin embedding by blocking using a stainless-steel brain block. Microtomy was performed at 10 μm, and sections were mounted on charged polyionic slides (Superfrost-plus, Fisher Scientific). For immunohistochemical analysis, deparaffinized tissue sections were incubated with primary antibody for identification of microglia [rabbit polyclonal anti-Iba1 (Wako Chemicals; 1:500). The secondary antibody was biotinylated rabbit IgG (for Iba1, 1:1000). A VIP kit (Vector Labs) reaction was used to yield a purple (Iba1) color. For neuronal analysis, hematoxylin and eosin (H&E) staining was performed (Diagnostic Laboratory, CSU). Stained slides were imaged using an Olympus SLIDEVIEW™ VS200 scanning microscope (Evident, Waltham, MA, USA) using an Olympus UPLXAPO40X (NA 0.95) objective (0.137 μm/pixel) equipped with a Hamamatsu ORCA-Fusion Digital CMOS camera (C14440-20UP, Hamamatsu Photonics, Shizuoka, Japan). Brightfield images were collected using Olympus VIA software in special mode, with the same imaging parameters utilized across each dataset.

### 2.3 Quantification of neuronal density using deep learning

To assess the number of healthy and pyknotic neurons in the dentate gyrus, custom neural networks were established to identify neurons using deep learning and trained for approximately 250,000 iterations across all experimental groups. Well-established histological standards (Mandour et al., 2021; Su et al., 2023; Xiong et al., 2025; Zhang et al., 2013) were followed to identify neurons based on nuclear size, pigmentation, and speckling. Pyknosis was determined by applying an eosinophilic threshold and nuclear condensation of identified neurons (Visiopharm Oncotopix, 2025.08.2). Networks were trained on 40X montage images of the hippocampus from 2-4 sections per animal (n = 3 animals/group) collected using an Olympus UPLXAPO40X (NA 0.95) objective (0.137 μm/pixel). Pre-defined regions of interest (ROIs) were drawn for the hippocampal subregions of the CA1 and CA3 pyramidal layers, and the dentate gyrus granular layer, using common anatomical landmarks referenced in the Allen Brain Atlas. Following established methods (Taborda-Bejarano et al., 2025; Green et al., 2022; Reddaway et al., 2023), photomicrographs were analyzed with each slide representing an independent observation for neuron counts. This approach allows greater sensitivity towards detection of subtle changes which were investigated in the present study.

### 2.4 Quantification of microglia identification and morphological assessment

To quantify microglial density and morphological features of reactivity, we applied neural networks trained with deep learning to identify microglia over approximately 400,000 iterations across all experimental groups, using 2-4 sections per animal (n = 3 animals/group). All network training was conducted in a supervised manner with performance checks occurring every 25,000 iterations. Microglial parameters were determined by assessing Iba1^+^ cells with wide-field 40X montage Z-stack deconvolved images (7.59 μm total depth, 1.75 μm step size), which was empirically determined to achieve optimal resolution to assess individual cell morphology (Green et al., 2022; Morrison et al., 2017). All scans were imported into Visiopharm, where a separate set of networks were used to skeletonize Iba1^+^ staining for each cell. The following parameters were assessed for each group: soma area, filament area, filament length, cellular perimeter, number of branchpoints, and number of endpoints. Quantitative phenomic analysis of cells was conducted using the Visiopharm Phenoplex™ plug-in to bin cells by reactive phenotypes. Photomicrographs were analyzed independently to increase sensitivity to changes in microglial density (Taborda-Bejarano et al., 2025; Green et al., 2022; Reddaway et al., 2023). Utilizing published methods (Taborda-Bejarano et al., 2025; Green et al., 2022; Reddaway et al., 2023; Kim et al., 2024), microglia were analyzed at the individual cell level to detect subtle changes in morphology. This method is used to more accurately identify subtle changes in microglial morphology that are not detectable when pooling across entire imaging frames or animals due to heterogeneity in this cell type. Analyzing a large population of microglia in this context is a standard and established approach when performing single cell morphometric analysis.

### 2.5 Statistical analysis

All data are shown as mean ± SEM, unless otherwise noted in the respective figure caption. Values from each experimental group were subjected to outlier analysis and exclusion via ROUT (α = 0.05). Statistical analysis between groups was accomplished via two-way ANOVA with Tukey post-hoc correction. Necessary transformations were performed to achieve normality. All statistical analyses were performed using GraphPad Prism (version 10.1.1; GraphPad Software, San Diego, CA, USA).

## 3 Results

**FIGURE 1:**
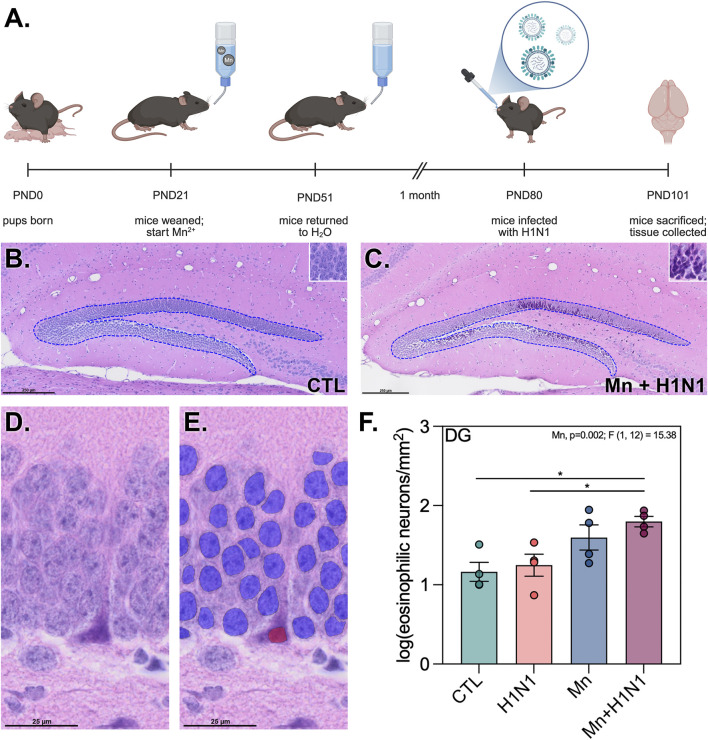
Manganese H1N1 co-treatment induces neuronal pyknosis in the dentate gyrus. **(A)** Study schematic highlighting the treatment paradigm employed to interrogate combined effects of Manganese (Mn) and Influenza A (H1N1) co-exposure on murine hippocampal histopathology. **(B,C)** Representative montage images of H&E stained untreated **(B)** and dual-treated **(C)** dentate gyri. **(D,E)** An eosinophilic neuron that is unlabeled **(D)** or labeled **(E)** by custom trained neural networks applying deep learning-based digital pathology. **(F)** Representative graphs depict the normalized number of eosinophilic neurons per mm^2^. *, **, ***, **** denote *p*-value <0.05, 0.01, 0.001, 0.0001, respectively, as measured using a two-way ANOVA. Treatment and interaction effects were tested for all conditions, with significant results reported. Bar graphs depict the mean and SEM. (*n* = 4 images/group; four slides/animal, three animals/treatment group). Study schematic was created using BioRender.com.

To assess if juvenile Mn exposure would potentiate the neuropathological effects of secondary adult H1N1 infection in the hippocampus, C57BL/6 mice were given water with or without Mn from PND21-51, then exposed to a sublethal dose of H1N1 or vehicle (Figure 1A). No weight or behavioral changes were observed during daily monitoring after viral infection. Following the application of high content microscopy and deep learning neural network image analysis, it was determined that Mn and H1N1 co-treatment resulted in significant increases in the number of pyknotic neurons in the dentate gyrus (Figures 1B–E). Figure 1F demonstrates the increase in the number of hypereosinophilic neurons with pyknotic features per mm^2^ between the control group and the dual-treated group (mean difference = 0.6348; p. = 0.0173), despite no change in the number of healthy neurons per mm^2^ from control to dual treatment (p. = 0.8553; Supplementary Figure S1A). Analysis of the CA3 pyramidal layer revealed a main effect from Mn (p. = 0.0163; F[1,12] = 7.793; Supplementary Figure S1C).

**FIGURE 2:**
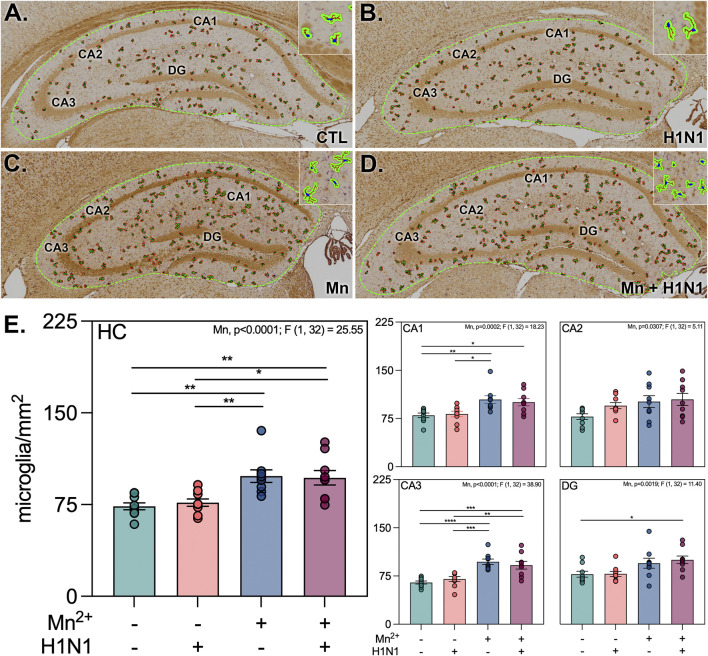
Treatment with manganese increases microglial density within the hippocampus. **(A–D)** Representative hippocampal montage images highlighting identified microglia across control **(A)**, H1N1 only **(B)**, Mn only **(C)**, and dual-treated **(D)** treatment groups. **(E)** Bar graphs depict the number of microglia per area for the whole hippocampus and each subregion. *, **, ***, **** denote *p*-value <0.05, 0.01, 0.001, 0.0001, respectively, as measured using a two-way ANOVA. Treatment and interaction effects were tested for all conditions, with significant results reported. Bar graphs depict the mean and SEM. (*n* = 9 images/group; three slides/animal, three animals/treatment group).

**FIGURE 3:**
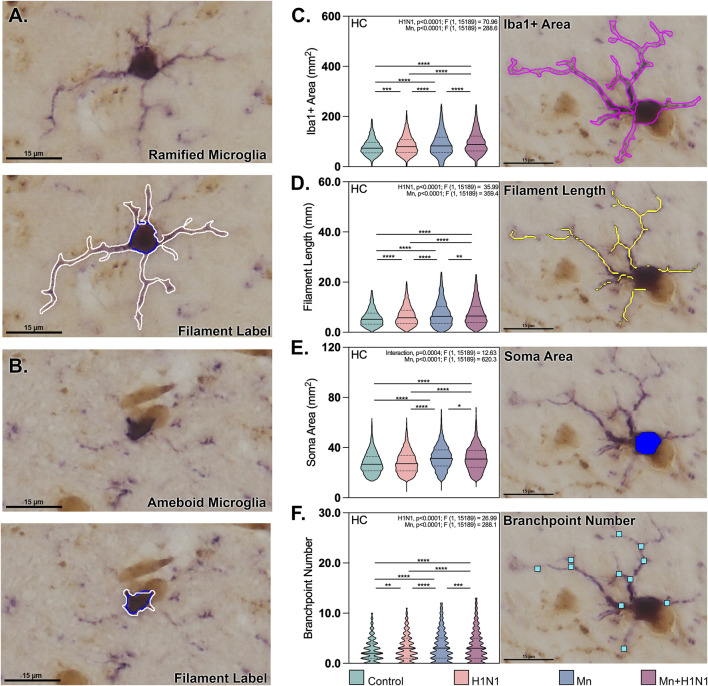
Microglial morphometric analysis reveals an increase in parameters indicating reactivity following manganese and influenza A treatments. **(A,B)** Representative high magnification scans depicting a ramified **(A)** and ameboid **(B)** microglia, with and without skeletonization mask. **(C–F)** Violin plots depict the Iba1^+^ area **(C)**, total filament length **(D)**, soma area **(E)**, and branchpoint number **(F)** for microglial populations across the entire hippocampal extent by treatment group. *, **, ***, **** denote *p*-value <0.05, 0.01, 0.001, 0.0001, respectively, as measured using a two-way ANOVA. Treatment and interaction effects were tested for all conditions, with significant results reported. Violin plots depict the mean, SEM, and total data distribution. (*n* = 3 slides/animal, three animals/treatment group).

We next assessed microglial reactivity across the hippocampus using deep-learning neural networks to evaluate morphometric changes in Iba1^+^ cells in four hippocampal subregions: Cornu Ammonis (CA) 1, 2, and 3, and the dentate gyrus (DG; Figures 2A–D). Mn exposure resulted in significantly increased microglial density per mm^2^ across the hippocampus in pairwise comparisons to control (Figure 2E; Mn mean difference = 23.34, p. = 0.0042; Mn + H1N1 mean difference = 24.69, p. = 0.0023), with no effect observed in animals only infected with H1N1. To interrogate whether parameters associated with microglial morphometry varied significantly by exposure type in this context, we generated a novel neural network capable of labeling Iba1^+^ filaments extending from individual identified somas (Figures 3A,B). All morphometric parameters were formally tested for pairwise and main effects, with only significant main effects reported (Figures 3C,D). Morphometric parameters were assessed for each cell independently, revealing that Mn treatment and H1N1 treatment both had main effects that significantly increased Iba1+ area per cell (Mn p. = <0.0001; F[1,15189] = 288.6; H1N1 p. = <0.0001; F[1,15189] = 70.93). A similar trend was observed for filament length (Mn p. = <0.0001; F[1,15189] = 359.4; H1N1 p. = <0.0001; F[1,15189] = 35.99) as well as branchpoint number (Mn p. = <0.0001; F[1,15189] = 288.1; H1N1 p. = <0.0001; F[1,15189] = 26.99) across the entire hippocampus (Figures 3C,D). Interestingly, somal area exhibited a Mn-specific main effect (p. = <0.0001; F[1,15189] = 620.3) and an interaction effect (p. = 0.0004; F[1,15189] = 12.63), but no H1N1-specific effect in these analyses. Combinatorial interaction effects were also observed in the CA1 and DG when assessing Iba1^+^ area (CA1 p. = 0.034; F[15,362] = 4.497; DG p. = 0.008; F[13,634] = 7.036). Separately, analysis of the CA3 subregion revealed an interaction effect by filament length (p. = 0.0007; F[15,416] = 11.59) and somal area (p. = 0.0012; F[15,416] = 10.46). These results indicate the importance of assaying the hippocampus region for subregion-specific dynamic responses (Supplementary Figure S2).

**FIGURE 4:**
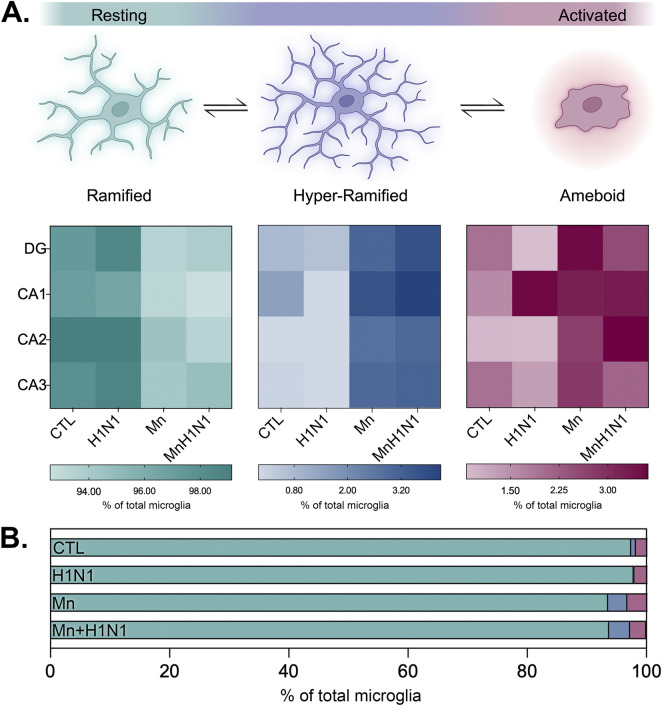
Microglial population phenomic analysis reveals a shift towards reactive morphologic states following exposure to manganese. **(A)** Heat maps depicting percentages of each reactive microglial phenotypes by subregion analyzed across the hippocampus. **(B)** Nested bar graph depicting reactive shifts in microglial population dynamics across the hippocampal extent. Cell representations were created using BioRender.com.

Given the diverse shifts observed in microglial size and shape by hippocampal subregion interest, we lastly employed the Phenoplex™ module (Visiopharm) to assess phenomic changes in microglia populations influenced by dual exposure to Mn and H1N1. Parameters used to bin and quantify cells into ramified, hyper-ramified, and ameboid cells were established based on the Iba1^+^ area, endpoints, and branchpoints per cell. Microglia were then sorted into each bin to provide region and treatment-specific population data. A heatmap was generated to visualize the relative percentage of each microglial subtype present per subregion (Figure 4A). Across all subregions, Mn treatment induced a shift in microglial reactivity towards a more activated state than vehicle or H1N1 (Figure 4B).

## 4 Discussion

PD is a devastating condition of largely unknown etiology, despite recent advances in our understanding of environmental toxicant-driven development of disease pathology (Dorsey and Bloem, 2024). Our group has previously shown that dual exposure to Mn and H1N1 does not result in significant neuronal loss in the SNpc but is associated with the development of a more reactive microglial phenotype in the basal midbrain (Bantle et al., 2021a). Given the increasing association of PD with dementia incidence and cognitive decline (Hely et al., 2008), we here sought to expand this investigation to include the effects of Mn priming and H1N1 infection on neuronal histopathology and microglial reactivity in the hippocampus.

In the present study, the total number of healthy neurons per mm^2^ in the granule cell layer of the dentate gyrus remained unchanged by treatment (Supplementary Figure S1A), so we generated sophisticated deep learning-based neural networks to investigate hypereosinophilic neurons with pyknotic nuclei, a more subtle indication of neuronal stress. Injured neurons are long understood to undergo chromatin condensation and cytoplasmic acidification during apoptotic and necrotic processes (Meisenholder et al., 1996; Hou et al., 2016). This acidification can be measured via eosin uptake to assess neuronal injury (Shen et al., 2016). We found an increase in the number of these eosinophilic neurons in dual-treated animals (Figure 1). This is especially noteworthy given the existence of literature linking apoptosis and necrosis with neurodegenerative diseases (Goel et al., 2022; Andreone et al., 2020; Moujalled et al., 2021). H&E staining is a standard, accurate methodology for assessing pathology in the central nervous system and identifying cell subtypes within the hippocampus (Mandour et al., 2021; Su et al., 2023; Xiong et al., 2025; Zhang et al., 2013). However, future studies could incorporate multi-plex immunostaining for additional verification of cell types affected in this model. Although the co-treatment did not result in overt neuronal loss at this time point, significant evidence of neuronal injury suggests an increased propensity for neurodegeneration at later time points following exposure and infection. Interestingly, this effect is driven by Mn, rather than an interaction between the challenges.

Microglia are known to plastically transition between reactivity states across the lifespan to modulate neuroinflammatory signaling and support neuronal health (Li and Barres, 2018; Hamagami et al., 2026; Kirkley et al., 2017). Still, certain immune modulatory insults have been shown capable of inducing aberrant activation of these cells, resulting in a transition from neurotrophic to neurotoxic outcomes (Blo et al., 2007). Here, we report that juvenile Mn exposure increases the number of microglia per mm^2^ in all four hippocampal subregions (Figures 2A–E), which is consistent with the glial-mediated inflammatory phenotype observed in LBD and other synucleinopathies (Loveland et al., 2023; Gomez-Nicola et al., 2013). Classically, reactive microglia are described as having an ameboid phenotype with short, thickened processes (Orihuela et al., 2016); however, a growing body of literature describes microglial activation as a continuum from resting (ramified) toward mildly activated (hyper-ramified) and activated (ameboid) morphologies (Holloway et al., 2019; Guo et al., 2022; Vidal-Itriago et al., 2022). We analyzed the morphology of microglia across the hippocampus to determine the morphometric phenotype of these cells following dual exposure to Mn and H1N1 (Figure 3). Cells in the co-treated brains showed increases in the Iba1^+^ area, filament length, and branchpoint number. Additionally, the average somal area increased, possibly indicating a hypertrophic phenotype, another activated state previously reported by other groups (Rao et al., 2024). One potential limitation of our existing analysis is the fact that peripheral monocytic cells, which can infiltrate the central nervous system, are known to express Iba1, which may contribute to shifts in the quantified parameters in this parenchymal population (Gonzalez Ibanez et al., 2019). This increased complexity of microglial cell morphology and reactivity suggests an important role of Mn in exerting an impact on microglial dynamics, with or without systemic infection.

Single-cell omics approaches have greatly increased our understanding of the heterogeneity of microglial responses on a molecular level (Ochocka and Kaminska, 2021). Manganese exposure has been shown capable of priming a lasting pro-inflammatory phenotype (Tjalkens et al., 2017; Moreno et al., 2009b) despite the mechanistic underpinnings of this remaining ill-explored. We similarly report a shift in the population reactivity of hippocampal microglia toward a reactive hyper-ramified phenotype following Mn treatment (Figure 4). This reactivity may result because Mn exposure has been shown capable of activating the cGAS-STING pathway in a non-nucleic acid-dependent manner (Zhang et al., 2025a; Zhang et al., 2025b). Mn has also been shown to stimulate the production of nitric oxide (Barhoumi et al., 2004; Moreno et al., 2008), which is known to exert neurotoxic effects when originating from microglia (Boje and Arora, 1992). Further, deletion of the *Nos2* gene has been shown to be protective against juvenile Mn exposure-induced motor dysfunction and dopaminergic denervation (Streifel et al., 2012). Still, the precise mechanisms that convey effects of juvenile immune priming into adulthood remain uncertain. Additional work from our group has shown that Mn-activated microglia amplify astrocyte inflammatory signaling (Kirkley et al., 2017). Astrocytes, another type of glial cell, are increasingly regarded as key players in the neurodegenerative sequelae with powerful, heterogeneous immune modulatory functions (Brandebura et al., 2023; Sofroniew, 2020; Rostami et al., 2020). Work from our group and others has also highlighted the important role that astrocyte inflammatory signaling holds in regulating α-synuclein aggregation and dopaminergic neuron loss after viral encephalitis (Bantle et al., 2021b). Mn exposure has also shown to be capable of disrupting calcium dynamics and inducing nitric oxide production in astrocytes, offering plausible routes of Mn-induced stress (Streifel et al., 2013; Liu et al., 2006). This work highlights the necessity for future efforts to better characterize the molecular mechanistic underpinnings associated with microglia-astrocyte crosstalk in this context.

Our present study utilized a two-hit model of juvenile Mn exposure and adult Influenza A infection to elucidate the impact of environmentally relevant exposures on neuronal health and microglial reactivity in the murine hippocampus. We found that Mn and the non-neurotropic H1N1 virus increased levels of neuronal stress and microglial activation without contributing to overt neurodegeneration. Our findings demonstrate the capacity of juvenile manganese exposure to induce a lasting reactive microglial phenotype in the hippocampus. Importantly, our data suggests that the effects of systemic immune insults may extend to the central nervous system parenchyma, contributing to a neuronal susceptibility to subsequent insults. This work highlights the need for future efforts to explore innate immunity and glial-mediated inflammatory signaling in the context of PD and related dementias.

## Data Availability

The raw data supporting the conclusions of this article will be made available by the authors upon reasonable request. Requests to access the datasets should be directed to ron.tjalkens@colostate.edu.
