# Supplementary material for: Exposure to manganese during juvenile development increases microglial activation in the hippocampus following systemic infection with A/California/04/2009 Influenza A H1N1 virus
Source: Front Toxicol. 2026 Apr 2;8:1789730. doi: 10.3389/ftox.2026.1789730 (PMC13082759; doi:10.3389/ftox.2026.1789730)
Supplement: Supplementary file 1 [file Supplementaryfile1.docx]

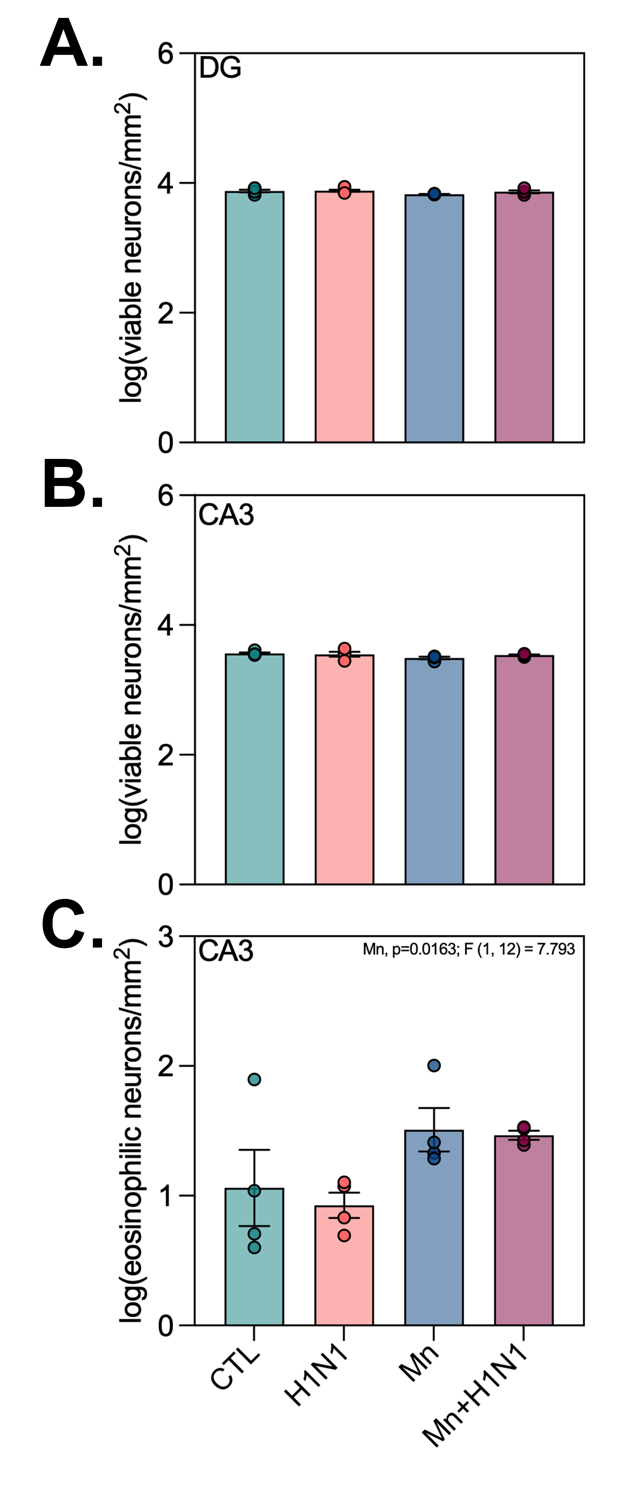


**Supplemental Figure 1. Manganese and H1N1 do not alter the number of viable neurons in the dentate gyrus or CA3.** Representative bar graphs depict the normalized number of viable neurons per mm^2^ in the dentate gyrus (A) or CA3 (B). Representative bar graphs depict the normalized number of eosinophilic neurons in the CA3 pyramidal layer. No significant differences were detected, as measured using a two-way ANOVA. Treatment and interaction effects were tested for all conditions, with significant results reported. Bar graphs depict the mean and SEM. (*n* = 4 images/group; 4 slides/animal, 3 animals/treatment group).
