# Supplementary material for: Exposure to manganese during juvenile development increases microglial activation in the hippocampus following systemic infection with A/California/04/2009 Influenza A H1N1 virus
Source: Front Toxicol. 2026 Apr 2;8:1789730. doi: 10.3389/ftox.2026.1789730 (PMC13082759; doi:10.3389/ftox.2026.1789730)
Supplement: Supplementary file 2 [file Supplementaryfile2.docx]

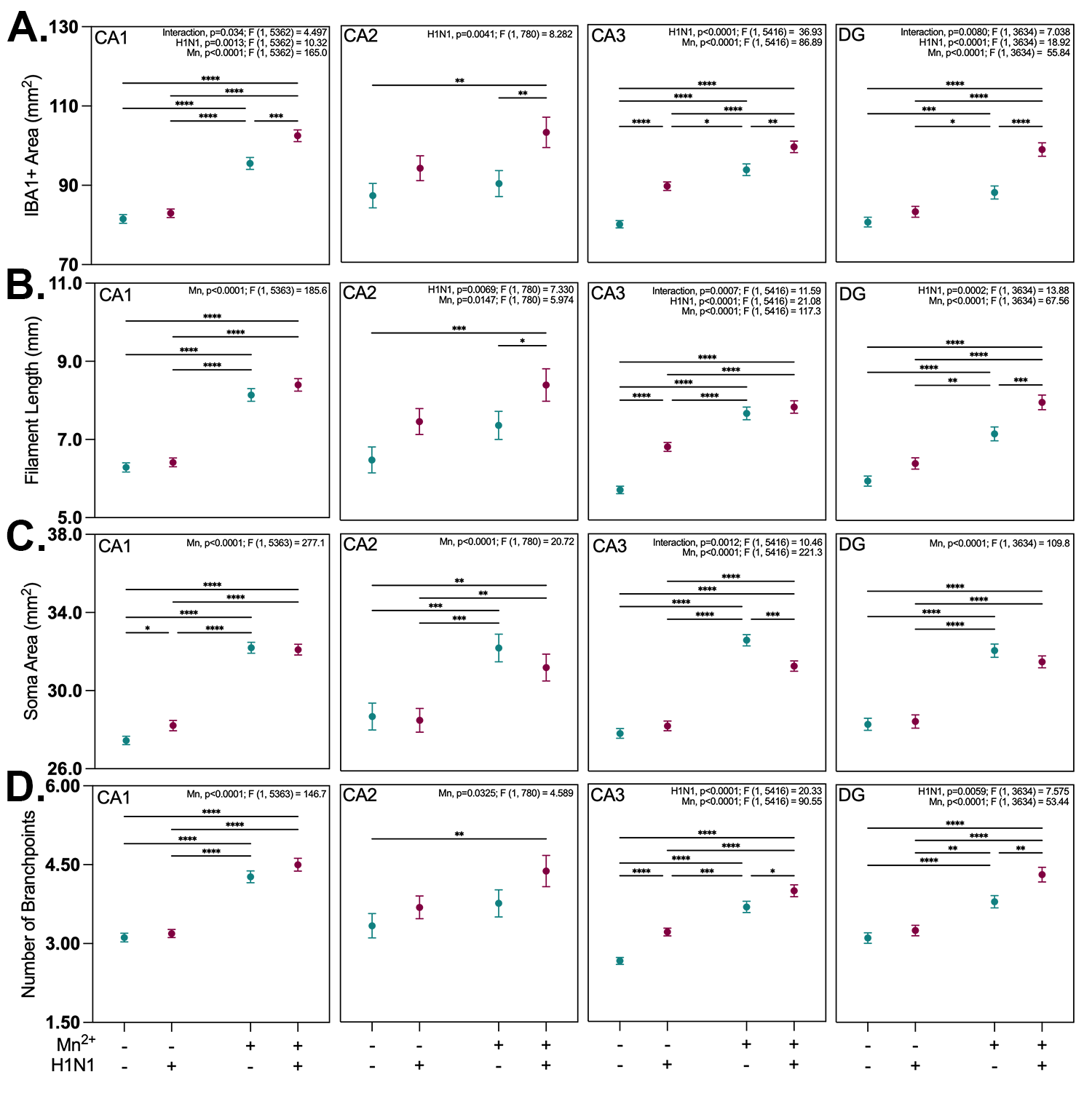


**Supplemental Figure 2. Region-specific microglial morphometric analysis reveals an increase in parameters indicating reactivity following manganese and influenza A co-treatment.** (A-D) Dot plots depict the Iba1^+^ area (A), total filament length (B), soma area (C), and branchpoint number (D) for microglial populations across each hippocampal subregion by treatment group. *, **, ***, **** denote *p*-value < 0.05, 0.01, 0.001, 0.0001, respectively, as measured using a two-way ANOVA. Treatment and interaction effects were tested for all conditions, with significant results reported. Dot plots depict the mean and SEM. (*n* = 9 images/group; 3 slides/animal, 3 animals/treatment group).
